# Retraction Note: Modulation of microglial phenotypes by dexmedetomidine through TREM2 reduces neuroinflammation in heatstroke

**DOI:** 10.1038/s41598-025-30133-y

**Published:** 2025-12-03

**Authors:** Ping Li, Tingting Shen, Xue Luo, Ju Yang, Zhen Luo, Yulong Tan, Genlin He, Zeze Wang, Xueting Yu, Ying Wang, Xuesen Yang

**Affiliations:** Department of Tropical Medicine, Army Medical University, Chongqing, China

Retraction of: *Scientific Reports* 10.1038/s41598-021-92906-5, published online 25 June 2021

The Editors have retracted this Article because of concerns regarding the figures presented in this work. An investigation conducted after its publication discovered the following issues:

• The *Tublin* bands in Figures 2A and 3D appear to overlap partially with each other;

• Panels in rows *Control* and *DEX* in Figure 2D appear to overlap partially with each other;

• Panels in row *DEX+Heat* in Figure 2D and row *DEX* in Figure 5A appear to overlap partially with each other;

• Panels in columns *Control* and *DEX* in Figure 3E appear to overlap partially with each other;

• Panels in columns *DEX* and *Heat + DEX* in Figure 3E appear to overlap partially with each other;

• Panels in columns *Heat *and *Heat + Dex* in Figure 3E appear to overlap partially with each other.

The data in question represent cells or tissues subject to different experimental conditions. The Editors therefore no longer have confidence in the integrity of the research presented in this Article.

The Authors did not reply to correspondence from the Editors about this retraction.

